# Cudraflavone B induces human glioblastoma cells apoptosis via ER stress-induced autophagy

**DOI:** 10.1186/s12868-023-00778-4

**Published:** 2023-01-31

**Authors:** Jinlin Pan, Rongchuan Zhao, Caihua Dong, Jiao Yang, Ruobing Zhang, Minxuan Sun, Nafees Ahmad, Yuanshuai Zhou, Yanxiang Liu

**Affiliations:** 1 School of Biomedical Engineering (Suzhou), Division of Life Sciences and Sciences and Medicine, University of Science and Technology of China, Hefei, 230026 China; 2 Jiangsu Key Lab of Medical Optics, Suzhou Institute of Biomedical Engineering and Technology, Chinese Academy of Sciences, Keling Road No.88, Suzhou, 215163 China; 3 Institute of Clinical Medicine Research, Suzhou Science & Technology Town Hospital, Suzhou, 215153 China; 4 Institute of Biomedical and Genetic Engineering, Islamabad, Pakistan; 5 Department of Pathology, Suzhou Science & Technology Town Hospital, No.1, Li Jiang Road, High-Tech District, Suzhou, 215153 China

**Keywords:** GBM, Endoplasmic reticulum stress, Unfolded protein response, Autophagy

## Abstract

**Background:**

Glioblastoma (GBM) is the most common malignant intracranial tumor with a low survival rate. However, only few drugs responsible for GBM therpies, hence new drug development for it is highly required. The natural product Cudraflavone B (CUB) has been reported to potentially kill a variety of tumor cells. Currently, its anit-cancer effect on GBM still remains unknown. Herein, we investigated whether CUB could affect the proliferation and apoptosis of GBM cells to show anti-GBM potential.

**Results:**

CUB selectively inhibited cell viability and induced cell apoptosis by activating the endoplasmic reticulum stress (ER stress) related pathway, as well as harnessing the autophagy-related PI3K/mTOR/LC3B signaling pathway. Typical morphological changes of autophagy were also observed in CUB treated cells by microscope and scanning electron microscope (SEM) examination. 4-Phenylbutyric acid (4-PBA), an ER stress inhibitor, restored the CUB-caused alteration in signaling pathway and morphological change.

**Conclusions:**

Our finding suggests that CUB impaired cell growth and induced cell apoptosis of glioblastoma through ER stress and autophagy-related signaling pathways, and it might be an attractive drug for treatment of GBM.

**Supplementary Information:**

The online version contains supplementary material available at 10.1186/s12868-023-00778-4.

## Background

GBM is the most common adult brain tumor (55%) with a high malignancy degree [1]. The common therapeutic strategies of GBM include surgery, radiation therapy, and chemotherapy [2]. Even though tremendous trials have been made to treat GBM [3, 4], the prognosis of GMB patients is still poor. The median survival time of newly diagnosed patients is less than 15 months.

Recently, several natural product-derived compounds including flavonoids have drawn more attention as potential agents for adjuvant GBM targeted therapies [5, 6]. The preliminary experiments suggest that natural products could harness the GBM cells via inducing apoptosis, increasing reactive oxygen species (ROS), inhibiting metastasis and regulating unfolded protein response (UPR)[7–9]. Moreover, the natural products, Apigenin-7-O-β-d-(-6′′-p-coumaroyl)-glucopyranoside (APG), caused severe and persistent ER stress in tumor cells leading to cell death [10, 11].

**Fig. 1:**
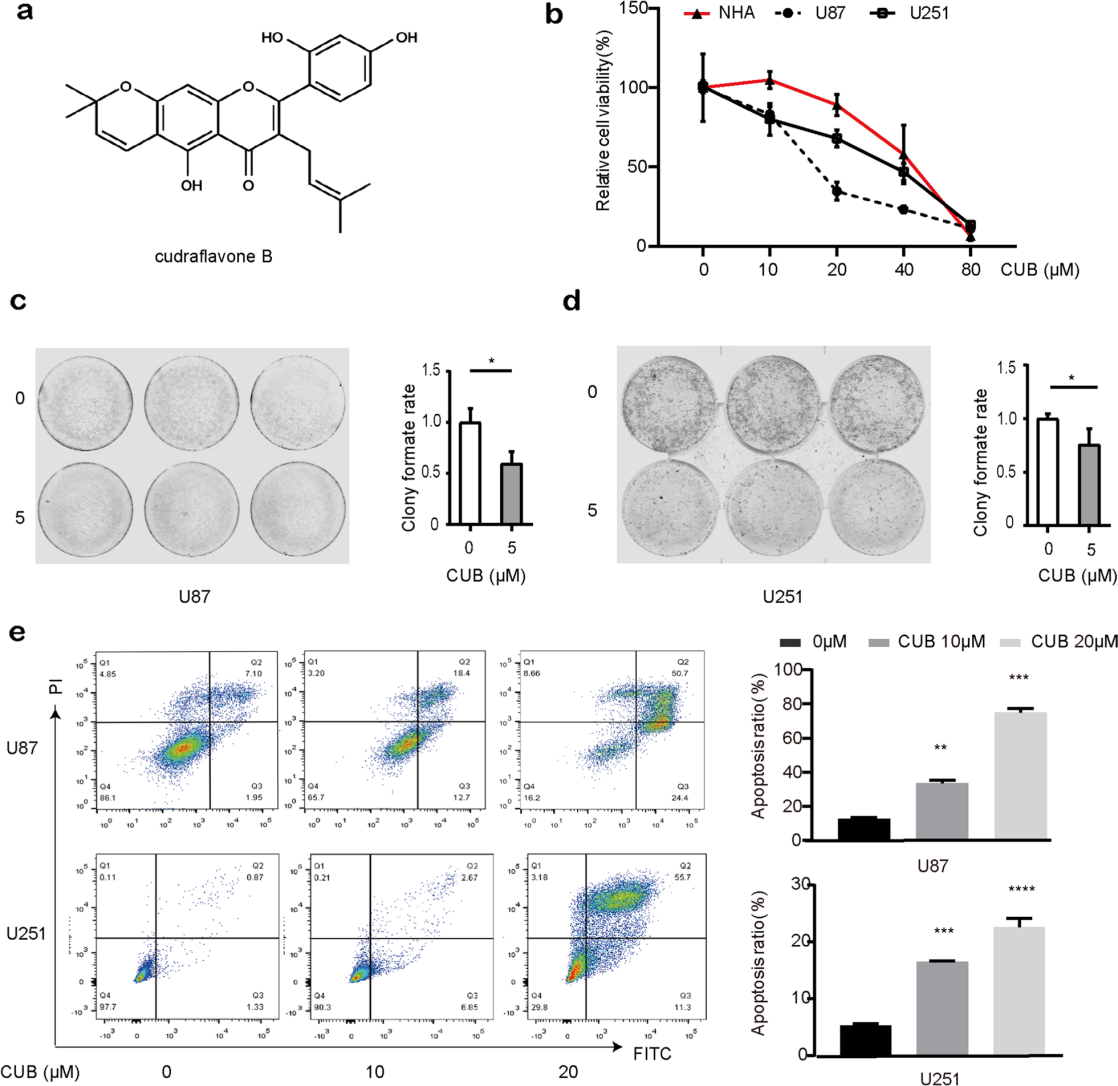
CUB increases apoptosis of GBM cells. **a** The structure of CUB. **b** The effects of CUB on cell viability of U87, U251 GBM cell lines and normal human astrocytes cells. **c**, **d** Clone formation assay of U87 **c** and U251 **d** cells treated with CUB. **e** Apoptosis measurements of GBM cells with CUB (10, 20 μM) incubation for 12(Additional file: Fig. S1a), 24 h. Data represents the mean ± SEM (*n* = 3). Statistical analysis was performed using one-way ANOVA. *P < 0.05, **P < 0.01, and ***P < 0.001

Cudraflavone B (CUB) is a flavonoid compound found in a variety of plants (Fig. 1a), first extracted and purified from Cephalotaxus fortune [12]. It has been applied to anti-aging in clinical due to its distinct antioxidant activity [13]. Furthermore, studies have shown that CUB induced apoptosis in human oral cancer cells and melanoma cells in vivo [14, 15]. In addition, CUB lead to a blockage of cell cycle sub-G1 and exhibited an inhibition effect on Monoamine oxidase (MAO) activity in mice [16]. All these findings indicated that CUB may have the potential to treat GBM. However, the experimental evidence is still lacking, and the underlying mechanisms remain elusive.

Based on these conditions, we engaged in the research for CUB in GBM. Here, we investigated whether CUB can affect the growth of GBM and explore the molecular mechanisms underlying its anti-GBM activity. We first tested the effect of CUB on the viability and proliferation in U87, U251 and normal human astrocyte cell lines. Next, transcriptome sequencing was used to identify the gene expression changes in CUB-treated cells. Finally, functional and mechanism experiments were conducted to further verify the molecular mechanisms underlying the anticancer effects of CUB in GBM.

## Materials and methods

### Cell lines and cell culture

Human GBM cell lines and normal human astrocytes(NHA), U87 and U251, were obtained from the Chinese Academy of Sciences Cell Bank (Shanghai, China). Cells were cultured in high glucose Dulbecco's modified Eagle's medium (DMEM) (Hyclone, USA) supplied with 10% fetal bovine serum (FBS) (Gibco, USA) and 100 μM streptomycin, 100 U/mL penicillin. All cells were cultured at 37 ℃ with 5% CO_2_ and 95% humidity in a constant temperature incubator. CUB (CUB, C25H24O6, Relative molecular mass: 420.5, purity > 94%, yellow powder) was purchased from WuXi PharmaTech (WuXi, China), which was dissolved in dimethyl sulphoxide (DMSO) at 30 mM as stored concentration and was diluted in DMEM for usage.

### Cell viability

Cell viability was detected using a cell-titer blue kit (Promega, USA). U87 and U251 cells (1 × 10^4^ cells/well) were seeded in 96-well plates and cultured for 24 h, then cells were treated with different concentrations of CUB for 48 h. To detect cell viability, 20 μl cell-titer blue reagents were diluted in 100 μl DMEM medium. In reverse experiments, cells were pre-treated with 4-PBA, 3-MA or CQ for 12 h before CUB treatment. Cell viability was quantified using Odyssey CLx Infrared Imaging System.

### Cloning formation assay

Cells were seeded in 6 well plates (1 × 10^3^ cells/well) and exposed to 5 μM CUB for 14 days. To stain the colony, cells were washed three times by PBS, then fixed with 4% polyformaldehyde (PFA) for 30 min and subsequently stained with 1% crystal violet for 5 min. Colonies were quantified using Odyssey CLx Infrared Imaging System.

### Apoptosis analysis

U87 and U251 cells were seeded in 6 well plates (4 × 10^5^ cells per well) and cultured for 24 h to achieve 90–100% confluence. After reagents treatment, annexin V-propidium iodide (AV-PI) staining was conducted to assess apoptosis rate following the manufacturer’s instructions (Beyotime, Shanghai, China). Cell apoptosis was detected by flow cytometry (FC) (BD Biosciences, AccuriTM C6; San Jose, CA, USA) and analyzed using the Flowjo software (Tree Star; Ashland, OR, USA).

### Western blot analysis

Cells were lysed in Radio Immuno Precipitation Assay (RIPA) buffer and the cell extraction was collected after centrifugation. Cell extraction was mixed with 5 × sodium dodecyl sulfate polyacrylamide gel electropheresis (SDS-PAGE) loading buffer (Beyotime, Shanghai, China), then boiled at 98 ℃ for 10 min. Proteins were separated by SDS–polyacrylamide gel electrophoresis then transferred to 0.22 μm Polyvinylidene Fluoride (PVDF) membranes (Millipore, Boston, MA, USA). Membranes were blocked for 2 h in Tris-buffered saline containing 5% non-fat dry milk (Biosharp, China), then cut it into several blots to hybridized with the indicated primary antibodies (1:1000 dilution) at 4 ℃ overnight. Horseradish peroxidase secondary antibody (1:2000) was incubated for approximately 2 h at room temperature then detected by the immobile western chemiluminescent horseradish peroxidase (HRP) substrate (Super ECL Detection, Yeasen, China) (ABclonal, China).

### Real-time PCR

RNA was extracted after 24 h post-drug treatment. The total RNA of samples was extracted using EZ-press RNA Purification Kit (EZBioscience, USA). A 4 × Reverse Transcription Master Mix (EZBioscience) was used for reverse transcription reaction at 42 ℃ for 15 min, 95 ℃ for 30 S. The 2 × SYBR Green qPCR Master Mix (EZBioscience) was used to perform qPCR following this protocol: denaturation (5 min, 95 ℃), and 40 amplification cycles (10 s at 95 ℃, and 30 s at 60 ℃) by using the Strata Gene Mx3000p (Agilent Technologies, Inc., Santa Clara, CA). Each gene of interest was normalized to glyceraldehyde phosphate dehydrogenase (GAPDH) and the fold change was compared relative to the control sample. Each assay was performed in triplicate and experiments were repeated in at least three pooled cell samples.

### SEM examination

Cells were fixed by 4% glutaraldehyde and postfixed in 1% OsO_4_ in 0.1 M cacodylate buffer for 2 h. After being stained with 1% Millipore-filtered uranyl acetate, the samples were then dehydrated in increased concentrations of ethanol, then infiltrated and embedded in epoxy resin (ZXBR, Spon 812). Electron photomicrographs of GBM cell ultra-structures were taken with a scanning electron microscope (JEM-1200EX II, JEOL; Tokyo, Japan).

### Transferase-mediated deoxyuridine triphosphate-biotin nick end labeling (TUNEL) analysis

TUNEL staining was performed using a TUNEL apoptosis detection kit (Servicebio, Wuhan). After CUB treatment, cells were immersed in 4% PFA solution for 15 min then washed with PBS. Next, proteinase K solution (20 μg/ml) was added for 30 min they washed with PBS. A 50-μl Equilibration Buffer was added to each sample for 10 min. Then added 56 μl TdT-labeled reaction mixture (the mixture was compounded: Recombinant TdT enzyme:CF640-dUTP Labeling Mix: Equilibration Buffer = 1 µl:5 µl:50 µl (1:5:50). The cells were placed in a wet box for 60 min at 37 °C then washed with Phosphate Buffered Saline (PBS). Images were taken under fluorescence microscopy (Leica, Japan) and five different fields were randomly selected. Three independent assays were conducted. The cell numbers were calculated using ImageJ-5.0 software (Windows, 64-bit Java 1.8.0_112). The TUNEL-positive rate equals the number of TUNEL-positive cells divided by the total cell number × 100%.

### Immunofluorescence (IF) staining

Immunofluorescence staining was performed in GBM cell lines growing in dishes of NEST^®^ cell culture (China). According to a standard protocol, ice-cold 4% PFA served as fixative. Primary antibodies were rabbit polyclonal Abs against human activating transcription factor 4 (ATF4) and C/EBP-homologous protein (CHOP) (Abclonal). Nuclei were stained with DAPI (4,6-diamidino-2′-phenylindol, 5 µg/ml). Secondary Abs were goat anti-rabbit Alexa Fluor 488 IgG and goat anti-mouse Alexa Fluor 647 (Invitrogen, Camarillo, CA).

### Transcriptome sequencing and expression analysis

U87 cells were treated with CUB then the total RNA was extracted as whole transcriptome libraries, and deep sequenced by Novogene Bioinformatics Technology Cooperation (Beijing, China). The protocol of differential expression analyses of genomics and Feature Counts of genomic features were described accordingly in previous study [17–20].

### mRFP-GFP-LC3 transfection

The adenovirus probe mRFP-GFP-LC3 was used to examine autophagic flux. The transfected cells were incubated with 4% paraformaldehyde. Autophagic flux was observed under Nikon microscope (Tokyo, Japan).

### Acridine orange (AO) staining

Cell apoptosis and lysosomal membrane permeability were evaluated by the AO staining assay (Sigma Aldrich, USA), when red fluorescence was emited in the acidic secondary lysosomes and diffuse green fluorescence in the cytoplasm [21]. Cells were incubated 5 μg/ml AO for 15 min, and observed under a Nikon microscope (Tokyo, Japan).

### Statistical analysis

The data were analyzed using GraphPad Prism 7.0. All data presented as means ± SEM. Statistical analysis was conducted by one-way analysis of variance (ANOVA). The statistical significances labeled as *P < 0.05, **P < 0.01, ***P < 0.001.

## Results

### CUB inhibited cell growth and induced apoptosis of GBM cells.

To determine the effect of CUB (Fig. 1a) on GBM cells, two GBM cell lines U87 and U251 were treated with different concentrations of CUB (5, 10, 20, 40, 80 μM) for 24 h. The Cell Titer-Blue results showed that GBM cells viability decreased significantly after CUB treatment, compared with normal human astrocytes (Fig. 1b). It also suggests that CUB suppressed cell survival in a dose-dependent manner, while the major differences between tumor cells and normal cells were observed at 10 and 20 μM in 24 h. In addition, cloning formation assays confirmed that CUB inhibited GBM cell survival ((Fig. 1c, d). Furthermore, we validated the CUB’s effect on apoptosis of the GBM cells. GBM cells were treated with CUB as indicated, then stained with Annexin V and PI for further flow cytometry analysis. CUB-induced cell apoptosis was found in a dose-dependent and time-dependent manner in both U87 and U251 cells (Fig. 1e, Additional file 1: Fig. S1a).

### CUB activated ER stress pathways in human GBM cells

**Fig. 2:**
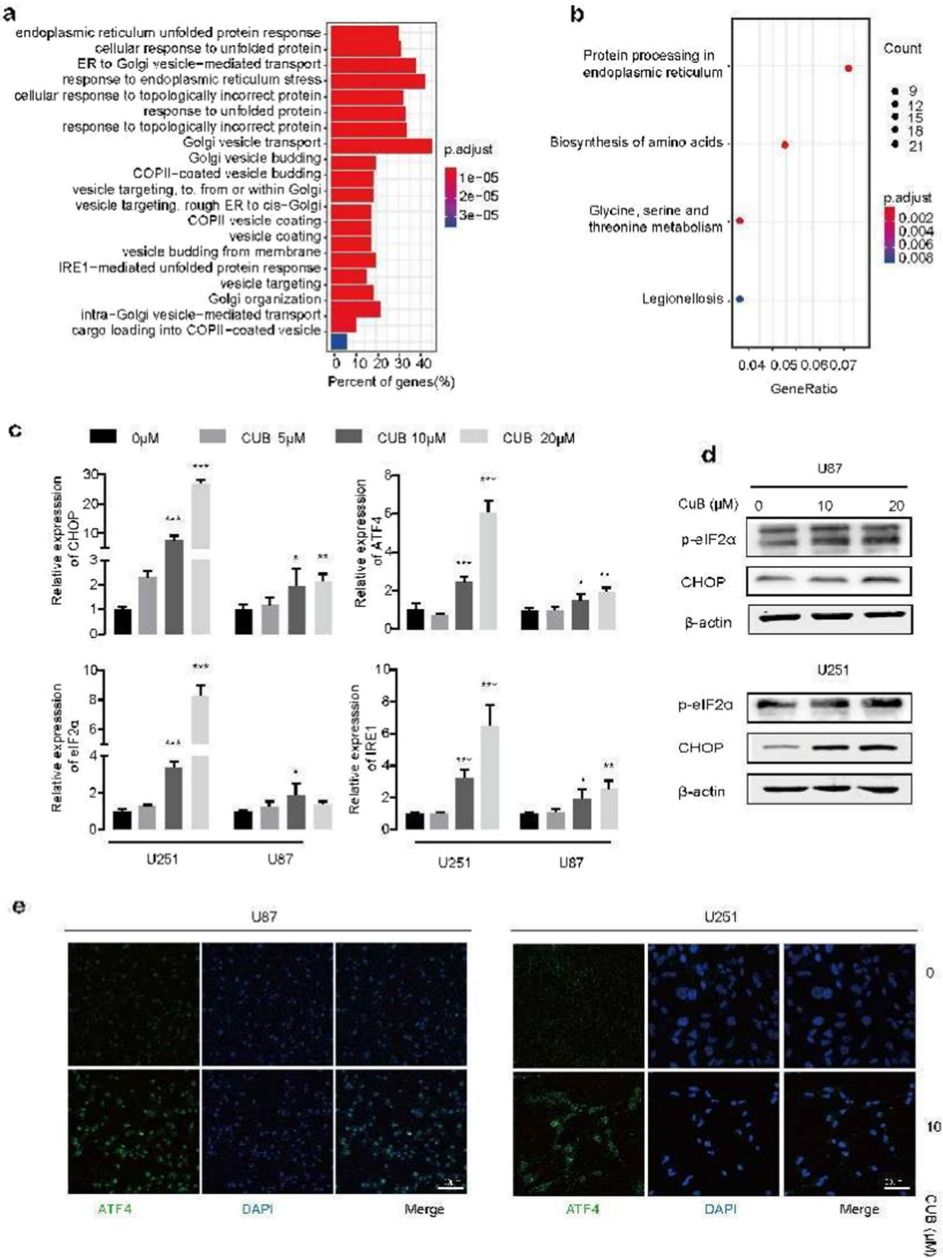
CUB activates ER stress. **a**, **b** Gene Ontology (GO) pathways **a** and Kyoto Encyclopedia of Genes and Genomes (KEGG) **b** analyzed the transcriptome sequencing of U87 GBM cells after CUB treatment. U87 cells were treated with 10 μM of CUB. **c** Quantitative real-time PCR (qPCR) analysis of GBM cells treated with of CUB (5,10, 20 µM) for 24 h. **d** Immunoblotting of the ER stress markers, p-eIF2Α and CHOP, in GBM cells (Notion: here are cropped blots, original cropped images and replicates are presented in Additional file 1: Fig. S2). **e** Fluorescence images of ATF4 in GBM cells exposed to CUB (10 μM) or control for 24 h. Data were mean ± SEM from three independent experiments. The statistical analysis was performed using one-way ANOVA, and the significance labeled as * P < 0.05, ** P < 0.01 and *** P < 0.001

Knowing that CUB could use to harness GBM cells, the underlying mechanism is still unknown. To address that, an RNA-seq analysis was employed. U87 cells were treated with 10 μM CUB for 24 h. Then the total RNA of treated and untreated cells was extracted for the transcriptome test. RNA-seq analysis revealed that the gene expression levels of ER stress and UPR pathways were significantly higher after CUB treatment (Fig. 2a, b). We next checked a group of ER stress-related genes. The expression level of phosphorylation of eukaryotic initiation factor-2α (eIF2α), CHOP and ER-stress sensor inositol-requiring enzyme 1(IRE1) were increased upon CUB treatment in a dose-dependent manner (Fig. 2c). To confirm this, we detected the ER stress-related protein level, CHOP and p-eIF2α. Indeed, it is very consistent with the result of real-time PCR (Fig. 2d).

In addition, immune fluorescence assays demonstrated that CUB cloud active ATF4 expression in U87 cells (Fig. 2e). In conclusion, our findings indicated that CUB triggers severe ER stress via PERK-eIF2α-ATF4 pathways.

### CUB induced autophagy and inhibited PI3K/mTOR/LC3B pathway

**Fig. 3:**
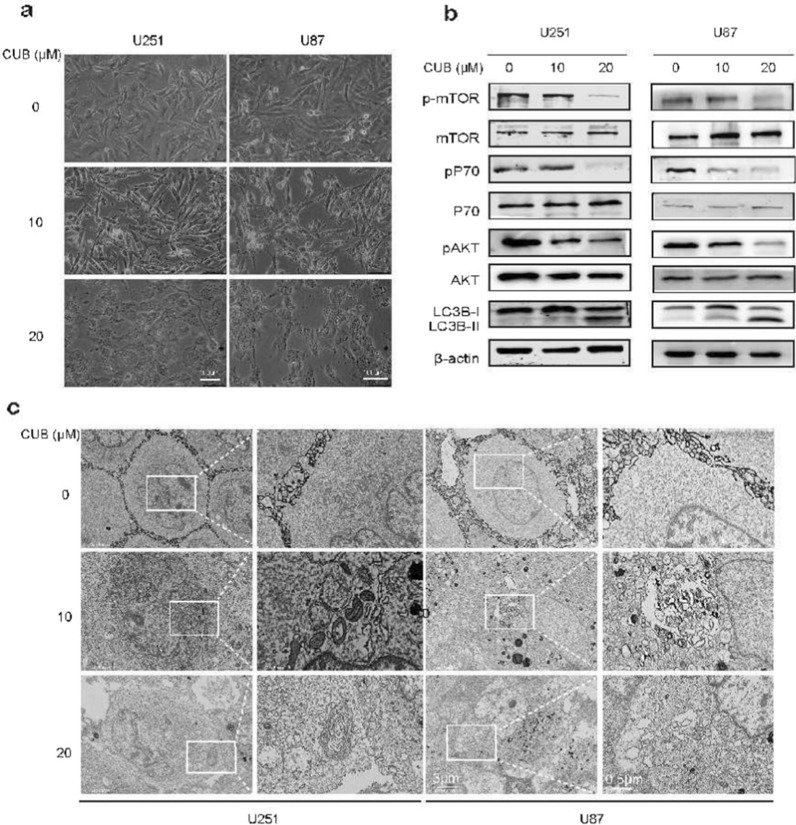
CUB induces GBM cell autophagy. **a** Morphological images of GBM cells. **b** Western blot analysis detected markers of the Akt/mTOR/pS70k pathway and LC3B-I, LC3-II in U251 cells treated with CUB at indicated concentration for 24 h (10 µg of the cell lysates was loaded)(Cropped blots, original cropped images and replicates are presented in Additional file 1: Fig. S5 and S6). **c** SEM images of U251, U87 cells treated with CUB (10, 20 µM) or DMSO for 24 h. Boxes highlight autophagic vacuoles and ER stress bubbles. Scale bars were marked in the images

ER stress often lead to a dramatic change of cell morphologies [22]. Indeed, after treatment with CUB for 24 h, GBM cells exhibited swelling, cytoplasmic vacuolization and cellular tentacles retraction (Fig. 3a). Among these, the cytoplasmic vacuoles accumulation is one of the typical characteristics of cell autophagy [23, 24]. To verify this, we used scanning SEM to characterize vacuole formation in U87 and U251 cells treated with 20 μM CUB for 12, 24 h. We observed the typical autophagy phenotype, the double-membrane structure of autophagic vacuoles and large cytoplasmic vacuoles on electron micrographs (Fig. 3c, Additional file 1: Fig. S1b). To further confirm whether CUB could cause autophagy in GBM cells, we performed mRFP-GFP-LC3 transfection assay and AO staining. According to the confocal fluorescence imaging, CUB treatment increased the LC3 II level in U87 and U251 cells, as indicated by increased GFP + mRFP + /GFP + mRFP- ratio and raised AO fluorescence intensity (Additional file 1: Fig. S3a, b), as well as increased ratio of LC3-II/ LC3I (Fig. 3b). Additionally, p-mTOR, p-p70 and p-Akt were down-regulated while mTOR, p70 and Akt were unchanged (Fig. 3b). Taken together, these data suggested that CUB activated the autophagy in GBM cells with classical morphological changes and harnessed PI3K/Akt/mTOR/LC3B signaling pathway.

3-Methyladenine(3-MA), PI3K inhibitor, was selected to inhibit the autophagy. To verify if ER stress-induced autophagy cause cell death, we performed the pre-treatment of 3-MA for 4 h to detect the autophagic flux and AO staining. We observed that 3-MA suppressed autophagic flux and prevented CUB-induced cell apoptosis (Additional file 1: Fig. S3a, b). To further verify CUB could induce autophagy-dependent cell death, we added another autophagy inhibitor chloroquine (CQ). CQ pretreatment reversed the inhibition of PI3K/Akt/mTOR pathway by CUB with increased phosphorylation levels of mTOR and P70 (Additional file 1: Fig. S4a) in U251 and U87 cells, and protected U87 cells from the cytotoxic effects of CUB (Additional file 1: Fig. S4b). Taken together, our results indicate that CUB promotes autophagy to induce cell death through inhibition of PI3K/AKT/mTOR signaling.

### 4-PBA could rescue the CUB triggered ER stress and autophagy-related phenotype

**Fig. 4:**
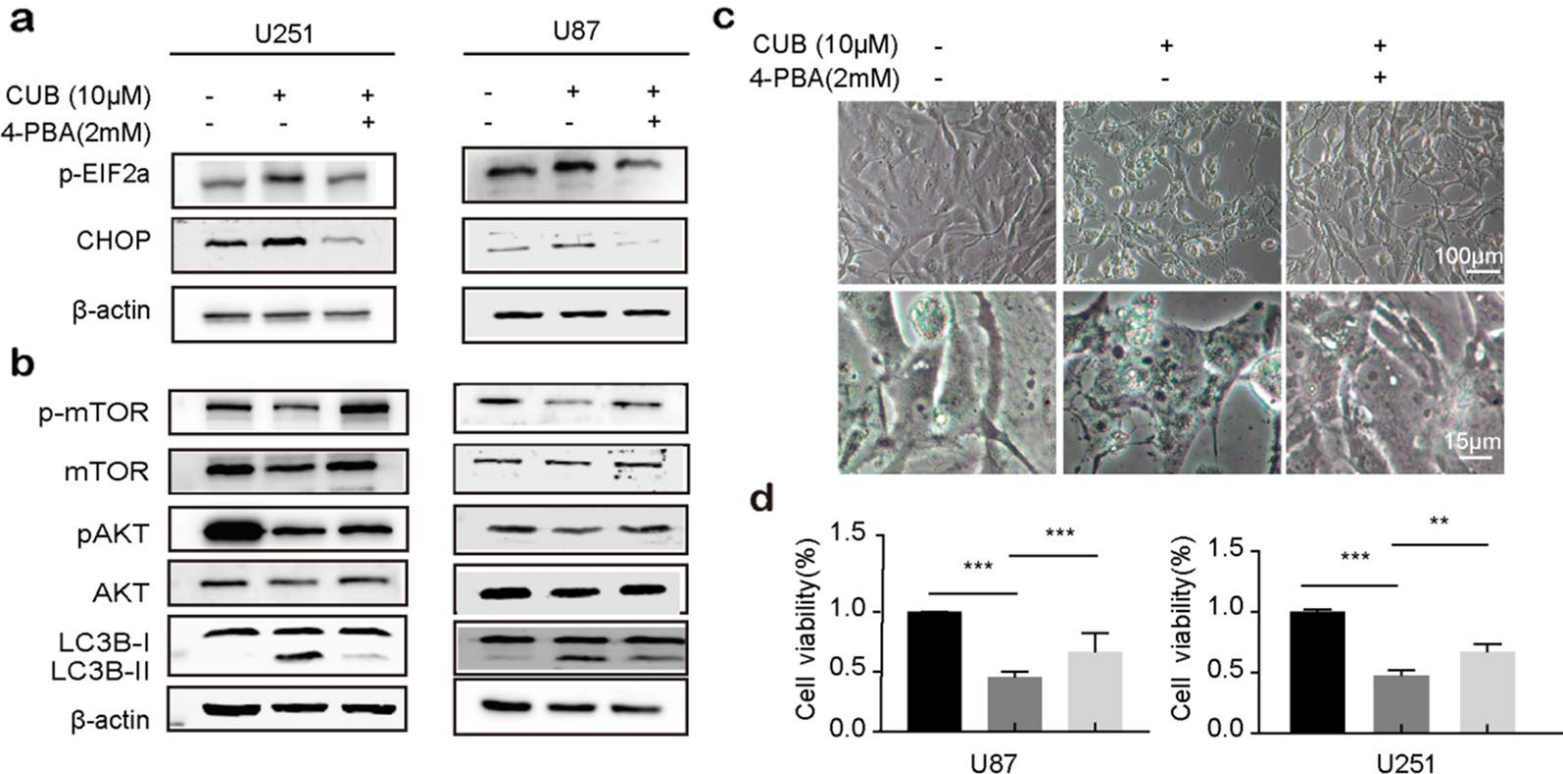
The ER stress inhibitor 4-PBA counteracts CUB. **a**, **b** Western blot detected the protein levels of p-eIF2Α, CHOP Akt/mTOR/pS70k pathway, LC3B, and β-actin in GBM cells pretreated with 4-PBA (2 mM) for 12 h before exposure to CUB (20 µM)(Cropped blots, original cropped images and replicates are presented in Additional file 1: Fig. S8 and. S9). **c** Morphological images of U87 cells treated with CUB (10 µM), 4-PBA (2 mM), and CUB (10 µM) with 4-PBA (2 mM) together, respectively (Cropped blots). **d** Cell viability of GBM cells CUB (10 µM), 4-PBA (2 mM) and CUB (10 µM) with 4-PBA (2 mM) together. The scale bars were marked in the image. All data were the mean ± SEM of values from experiments performed in triplicate. * P < 0.05, ** P < 0.01 and *** P < 0.001 compared to control

To better understand the roles of ER stress signaling in CUB-mediated ER stress and autophagy, we investigated whether abolish the ER stress-related pathways could reverse the CUB-mediated phenotype. The GBM cells were pre-treated with 4-PBA (an ER stress inhibitor) for 12 h before CUB treatment. Then, we checked the ER stress- and autophagy-related protein levels. As expected, the ER stress inhibitor 4-PBA counteracted the expression change of p-eIF2α, CHOP and Akt/mTOR/LC3B caused by CUB (Fig. 4a, b). Correspondingly, ER stress vacuoles reduced distinctly in 4-PBA pretreated cells (Fig. 4c). In addition, 4-PBA increased the viability of GBM cells, which was decreased by CUB treatment (Fig. 4d). To further verify the effect of 4-PBA on cell apoptosis, we conducted TUNEL assay. In line with cell viability results, 4-PBA counteracted CUB in apoptosis (Additional file 1: Fig. S7a). These observations confirmed that CUB-induced apoptosis via activating the ER stress related pathways.

## Discussion

Natural products produced from the plants are important sources of screening leading compounds in cancer treatment. To date, more than half of the FDA-approved anti-tumor drugs come from natural products and their derivatives [25]. In this study, we revealed that natural flavonoid CUB suppressed cell growth and promoted cell apoptosis in GBM. Moreover, we investigated the underlying molecular mechanisms, and found that CUB stimulates ER stress-dependent autophagy in human GBM cells via the PERK/eIF2 pathway. Inhibition of CUB-induced ER stress can prevent autophagy and restored cell vitality. Together, these data suggested that CUB is a potential drug candidate for GBM therapy.

Temozolomide (TMZ) was used to treat GBM for over a decade, but its treatment benefits are limited due to resistance [26]. CUB has been reported to show anti-proliferative activity in B16 melanoma cells, human gastric carcinoma cells and human oral cancer cells with effective lowest concentration at the 12uM in previous experiments. Our studies showed that CUB induced cell apoptosis in both U87 and U251 GBM cells by ER stress-induced autophagy, here we showed that its IC50 was 10uM, which indicated it is promising for in vivo experiments and clinical studies.

In our study, CUB induced ~ 56% cell death at the dose of 20uM after 24 h in U87 cells, while ~ 40% cell death in the case of U251(Fig. 1a). We found U87 displayed earlier response to CUB- induced ER stress as evidenced by the earlier appearance of intracellular vacuoles after 12 h treatment of CUB (Additional file 1: Fig. S1c). Previous research reported that although U87 and U251 cells showed high ratios of PI3K-AKT signaling pathway activation, U251 cells are more resistant to hypoxia and low glucose condition [27]. The glycolysis rate of cells plays a vital role in mitochondrial respiration function to determine the relative contribution of autophagy, which is consistent with the lower autophagy level that was observed for U251 cells compared with U87 cells in another study (Additional file 1: Fig. S1c). This may reflect that U251 cells has a slowly autophagy response to ER stress induced by CUB.

Although CUB shows promising anticancer-activity in multiple cancers types via inducing apoptosis, our knowledge of its underlying molecular mechanisms is very limited, e.g. regulating the tumour necrosis factor α (TNFα) and nuclear fractor-κB (NF-κB) expression, affecting the anti-inflammatory response [28], and activating the MAPK signaling pathway to promote apoptosis [13]. Here, we observed ER stress was induced by CUB treatment in GBM cells. Based on transcriptome analysis results, we demonstrated that the CUB-induced GBM cell apoptosis is dependent upon persistent ER stress that activates the PERK/ATF4/CHOP pathway. It is very well in line with that persistent ER stress causes tumor apoptosis, provides prospects for tumor treatment strategies [22, 29]. In addition, we also observed cell cycle inhibition upon CUB. This is consistent with a previous study that in response to PERK-ATF4-CHOP UPR reaction, eIF2α is activated to inhibit cyclin-D1 expression, causing cell cycle G1 blocking [30].

Furthermore, our results support that drug-induced persistent ER stress resulted in cell death via autophagy [31]. CUB treated cells showed the classic morphological and molecular characteristics of autophagy, such as autolysosomes formation and the membrane-binding LC-II. [32]. Mechanically, the result highlighted CUB-induced ER stress-activated autophagy through Akt/mTOR/RPS6KB1 pathway. In line with this, the ER stress inhibitor increased the activity of Akt/mTOR/RPS6KB1 signaling, leading to less autophagy flux. These results highlighted this pathway as a potential mediator of CUB-induced autophagy in GBM cells. Considering the dysregulated and reprogrammed metabolic environment in cancer cells, it needs deep understanding for the PERK/ATF4/CHOP in crosstalk between ER stress and apoptosis.

However, inhibition of ER stress does not fully abrogate cell death. This may be because excess CUB impairs cell viability through activation of NF-κB (Additional file 1: Fig. S7b). Additionally, it has shown impressive anti-inflammatory and neuro-protective effects on macrophages. In GBM, tumor-associated macrophages comprise half of tumor mass and involving in tumor progression [33]. We believe that it will be worth further exploration through its promotion effect on macrophages and resistance to tumors, if it can cross the blood–brain barrier. Currently, the concentrations of CUB we used are close to those used in other anti-tumor reports. However, we did not conduct in vivo experiment to assess its permeability to the blood–brain barrier and suitable administration concentration. We insight it will be further explored in the next studies.

## Supplementary Information

**Additional file 1. Fig. S1.** CUB induced autophagy through ER stress-dependent pathways. **(a)**Flow cytometry analysis results of U87, U251 cells at 12h treatment of CUB (10μM, 20μM). **(b)** Representative SEM images of U87, U251 cells occurred autophagy induced by CUB (10μM, 20μM).** (c)**Morphological images of GBM cells at 6h of CUB treatment. n = 3 per group. Means ± SD. *P < 0.05, **P < 0.01, ***P < 0.001. **Fig. S2.** CUB activates ER stress, blots origin images and replicates of Fig2d. **Fig. S3.** CUB activates autophagy flux in GBM cells. **(a)** Confocal images and the ratio of GFP-LC3/mRFP-LC3 at 12h. CUB (10μM), 3-MA(10mM). **(b)** Confocal images of AO staining at 12h. **Fig. S4.** CUB induced cell death through autophagy-dependent pathways. **(a)** Western blotting analysis of U87, U251 cells at 12h treatment of CUB (0μM, 20μM) or 3-MA(1mM) and CQ(30μM) pretreatment followed treatment of CUB(20μM). **(b)** Autophagy inhibitors suppressed U87 cell proliferation detected by the Cell-title Blue assay. Cells were incubated with 3-MA(1mM) or CQ (30μM) 12 h and then cultured in CUB(20μM) medium for the indicated times. Means ± SEM. *P < 0.05, **P < 0.01, ***P < 0.001. **Fig. S5. **CUB induces GBM cell autophagy, blots origin images and replicates of Fig. 3b (U87). **Fig. S6.** CUB induces GBM cell autophagy, blots origin images and replicates of Fig. 3b (U251). **Fig. S7.** CUB promoted apoptosis and NF-κB activation in U87 cells.** (a) **Images of TUNEL staining Green: TUNEL-positive cells, Blue: DAPI. **(b)** Western Blot analysis of NF-κB pathway. All data are expressed as the mean ± SEM of values from experiments performed in triplicate. * P < 0.05, ** P < 0.01 and *** P < 0.001 compared to control (Cropped blots). **Fig. S8.** The ER stress inhibitor 4-PBA counteracts CUB, blots origin images and replicates of Fig. 4a. **Fig. S9.** The ER stress inhibitor 4-PBA counteracts CUB, blots origin images and replicates of Fig. 4a. **Fig. S10.** CUB promoted apoptosis and NF-κB activation in U87 cells. blots origin images and replicates of Fig. S1b. **Fig. S11.** Part images of differential contrast blot

## Data Availability

The datasets generated during and analyzed during the current study are not publicly available due to we propose to continue our study of this drug in GBM therapy but are available from the corresponding author on reasonable request.

## Abbreviations

GBM: Glioblastoma
CUB: Cudraflavone B
APG: Apigenin-7-O-β-d-(-6″-p-coumaroyl)-glucopyranoside
UPR: Unfolded protein response
ROS: Reactive oxygen species
4-PBA: 4-Phenylbutyric acid
MAO: Monoamine oxidase
NHA: Normal human astrocytes
FBS: Fetal bovine serum
DMSO: Dimethyl sulphoxide.
FC: Flow cytometry
RIPA: Radio immuno precipitation assay
SDS-PAGE: Sodium dodecyl sulfate polyacrylamide gel electropheresis
PVDF: Polyvinylidene fluoride
HRP: Horseradish peroxidase
GAPDH: Glyceraldehyde phosphate dehydrogenase
TUNEL: Transferase-mediated deoxyuridine triphosphate-biotin nick end labeling
PBS: Phosphate buffered saline
IF: Immunofluorescence
CHOP: C/EBP-homologous protein
IRE1: ER-stress sensor inositol-requiring enzyme 1
eIF2α: Eukaryotic initiation factor-2α
KEGG: Kyotoe ncyclopedia of genes and genomes
GO: Gene ontology
qPCR: Quantitative real-time PCR
TMZ: Temozolomide
TNFα: Tumour necrosis factor α
CQ: Chloroquine
