# Supplementary material for: Cudraflavone B induces human glioblastoma cells apoptosis via ER stress-induced autophagy
Source: BMC Neurosci. 2023 Jan 31;24:10. doi: 10.1186/s12868-023-00778-4 (PMC9890863; doi:10.1186/s12868-023-00778-4)
Supplement: Supplementary file 1 — Additional file 1. Fig. S1. CUB induced autophagy through ER stress-dependent pathways. (a)Flow cytometry analysis results of U87, U251 cells at 12h treatment of CUB (10μM, 20μM). (b) Representative SEM images of U87, U251 cells occurred autophagy induced by CUB (10μM, 20μM). (c)Morphological images of GBM cells at 6h of CUB treatment. n = 3 per group. Means ± SD. *P < 0.05, **P < 0.01, ***P < 0.001. Fig. S2. CUB activates ER stress, blots origin images and replicates of Fig2d. Fig. S3. CUB activates autophagy flux in GBM cells. (a) Confocal images and the ratio of GFP-LC3/mRFP-LC3 at 12h. CUB (10μM), 3-MA(10mM). (b) Confocal images of AO staining at 12h. Fig. S4. CUB induced cell death through autophagy-dependent pathways. (a) Western blotting analysis of U87, U251 cells at 12h treatment of CUB (0μM, 20μM) or 3-MA(1mM) and CQ(30μM) pretreatment followed treatment of CUB(20μM). (b) Autophagy inhibitors suppressed U87 cell proliferation detected by the Cell-title Blue assay. Cells were incubated with 3-MA(1mM) or CQ (30μM) 12 h and then cultured in CUB(20μM) medium for the indicated times. Means ± SEM. *P < 0.05, **P < 0.01, ***P < 0.001. Fig. S5. CUB induces GBM cell autophagy, blots origin images and replicates of Fig. 3b (U87). Fig. S6. CUB induces GBM cell autophagy, blots origin images and replicates of Fig. 3b (U251). Fig. S7. CUB promoted apoptosis and NF-κB activation in U87 cells. (a) Images of TUNEL staining Green: TUNEL-positive cells, Blue: DAPI. (b) Western Blot analysis of NF-κB pathway. All data are expressed as the mean ± SEM of values from experiments performed in triplicate. * P < 0.05, ** P < 0.01 and *** P < 0.001 compared to control (Cropped blots). Fig. S8. The ER stress inhibitor 4-PBA counteracts CUB, blots origin images and replicates of Fig. 4a. Fig. S9. The ER stress inhibitor 4-PBA counteracts CUB, blots origin images and replicates of Fig. 4a. Fig. S10. CUB promoted apoptosis and NF-κB activation in U87 cells. blots origin images and repl [file 12868_2023_778_MOESM1_ESM.docx]

**Additional file Information**

**Cudraflavone B induces human glioblastoma cells apoptosis via ER stress-induced autophagy**

Jinlin Pan^1,2^, Rongchuan Zhao^1,2^, Caihua Dong^1,2^, Jiao Yang^3^, Ruobing Zhang^1^, Minxuan Sun^1^, Nafees Ahmad^4^, Yanxiang Liu^5, *^, Yuanshuai Zhou^1, *^

Corresponding author: Yuanshuai Zhou^1, *^

Email: [zhouys@sibet.ac.cn](mailto:zhouys@sibet.ac.cn)

**This Additional file information contains:**

- 15 Pages
- Additional file Materials and Methods
- Additional file Figures (11 Figures, S1 to S11)

**Additional file Materials and Methods**

**Western blot analysis**

Cells were lysed in Radio Immuno Precipitation Assay (RIPA) buffer and the cell extraction was collected after centrifugation. Cell extraction was mixed with 5× sodium dodecyl sulfate polyacrylamide gel electropheresis (SDS-PAGE) loading buffer (Beyotime, Shanghai, China), then boiled at 98℃ for 10 min. Proteins were separated by SDS-polyacrylamide gel electrophoresis then transferred to 0.22 μm Polyvinylidene Fluoride (PVDF) membranes (Millipore, Boston, MA, USA). Membranes were blocked for 2 h in Tris-buffered saline containing 5% non-fat dry milk (Biosharp, China), then cut it into several blots to hybridized with the indicated primary antibodies (1:1000 dilution) at 4℃ overnight. Horseradish peroxidase secondary antibody (1:2000) was incubated for approximately 2 h at room temperature then detected by the immobile western chemiluminescent horseradish peroxidase (HRP) substrate (Super ECL Detection, Yeasen, China).

**mRFP-GFP-LC3 transfection**

The adenovirus probe mRFP-GFP-LC3 was used to examine autophagic flux. The transfected cells were incubated with 4% paraformaldehyde. Autophagic flux was observed under Nikon microscope (Tokyo, Japan).

**Acridine orange (AO) staining**

Cell apoptosis and lysosomal membrane permeability were evaluated by the AO staining assay (Sigma Aldrich, USA), when red fluorescence was emited in the acidic secondary lysosomes and diffuse green fluorescence in the cytoplasm[[18](#_ENREF_18)]. Cells were incubated 5 μg/ml AO for 15 min, and observed under a Nikon microscope (Tokyo, Japan).

**Additional file Figures**

#
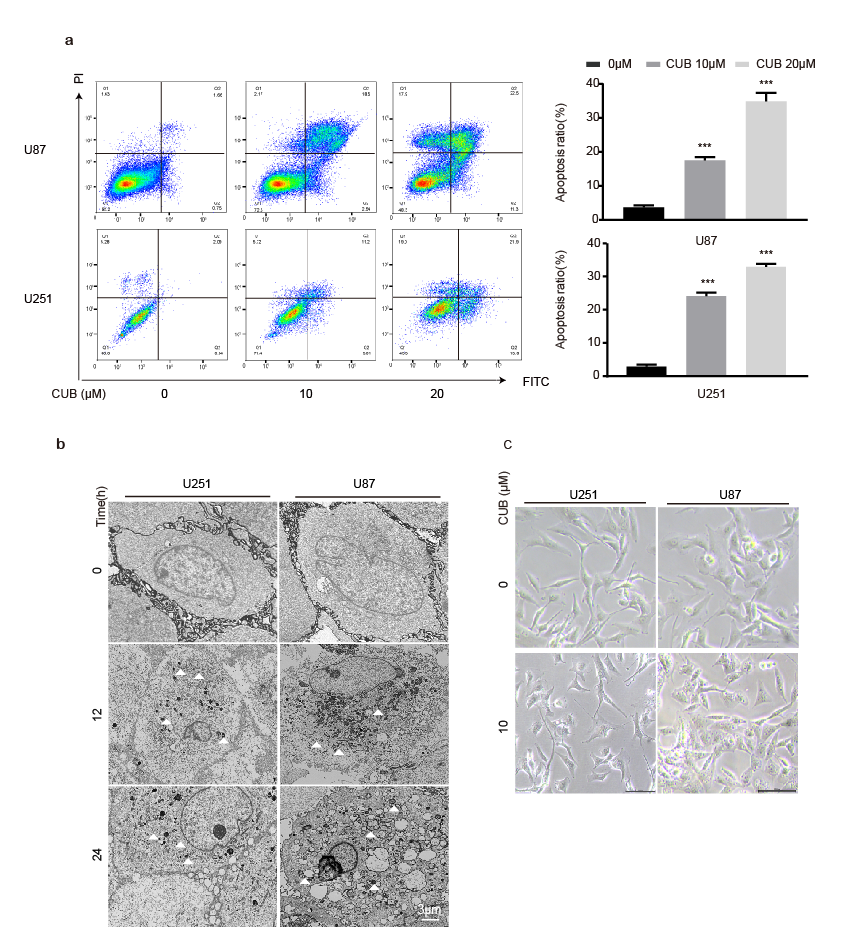


**Fig.S1 CUB induced autophagy through ER stress-dependent pathways**

**(a)**Flow cytometry analysis results of U87, U251 cells at 12h treatment of CUB (10μM, 20μM). **(b)** Representative SEM images of U87, U251 cells occurred autophagy induced by CUB (10μM, 20μM). **(c)**Morphological images of GBM cells at 6h of CUB treatment. n = 3 per group. Means ± SD. *P < 0.05, **P < 0.01, ***P < 0.001.

**
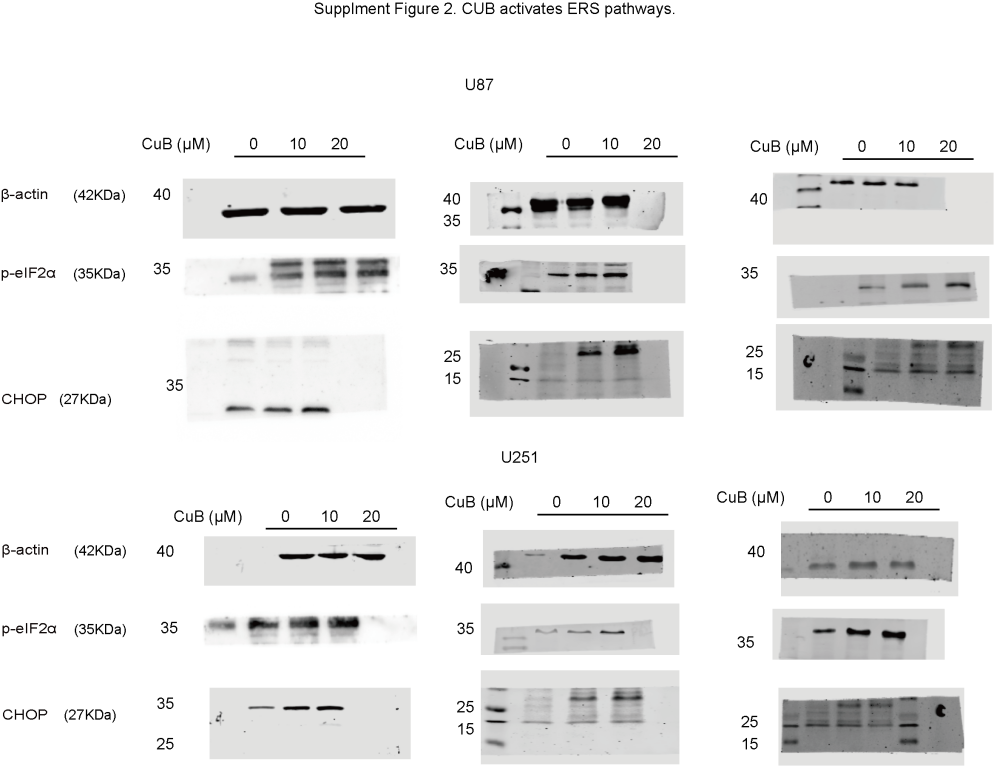
**

**Fig.S2 CUB activates ER stress, blots origin images and replicates of Fig2.d**

**
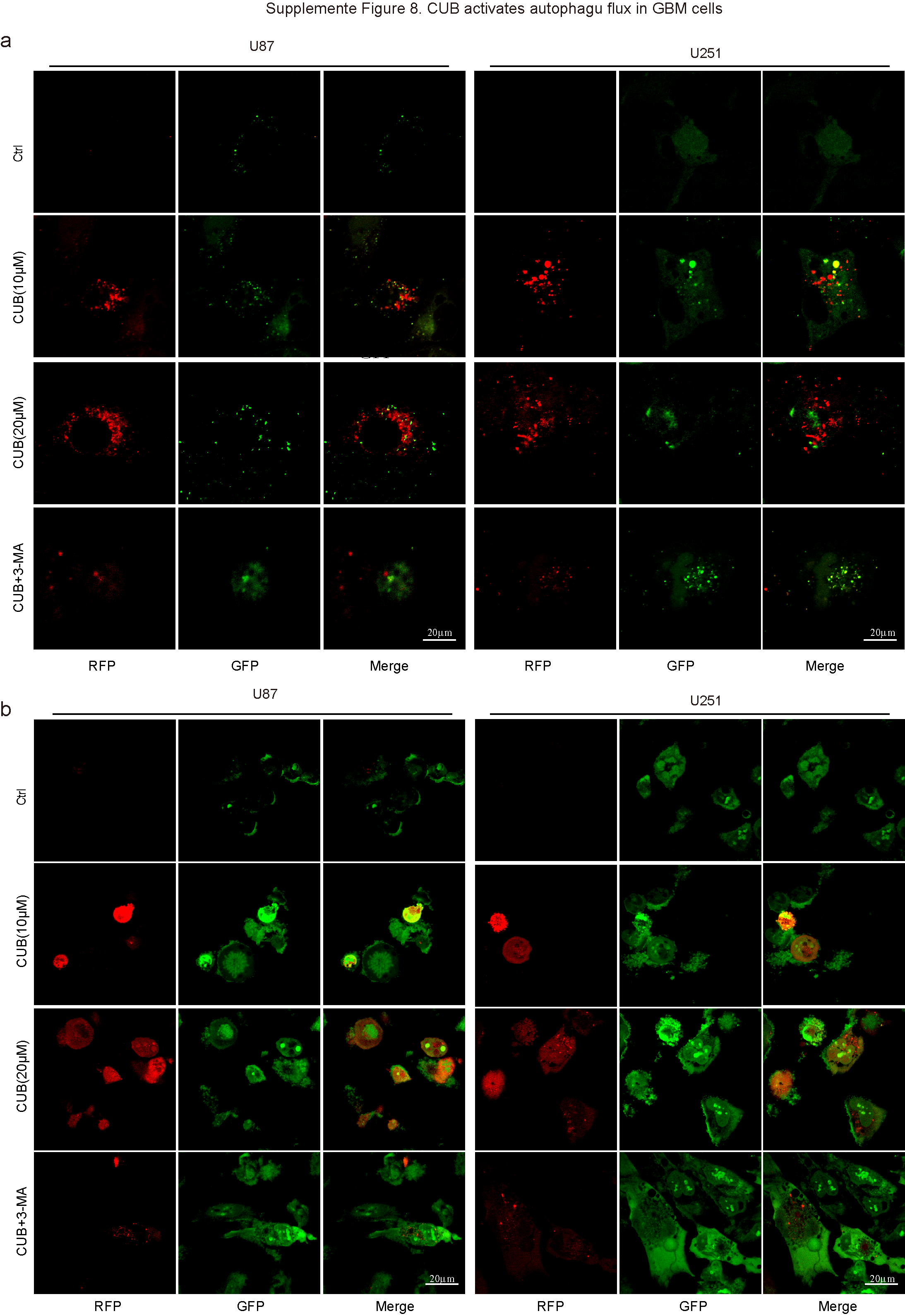
**

**Fig.S3 CUB activates autophagy flux in GBM cells**

1. Confocal images and the ratio of GFP-LC3/mRFP-LC3 at 12h. CUB (10μM), 3-MA(10mM). **(b)** Confocal images of AO staining at 12h.

**
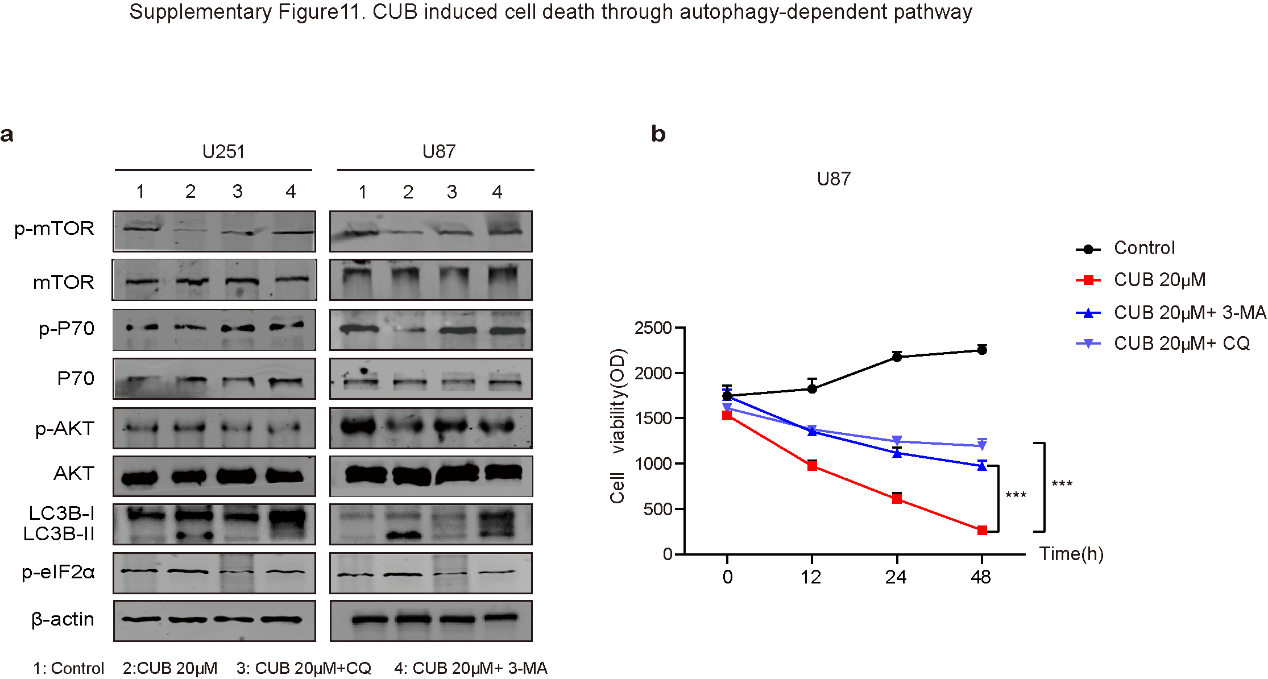
**

**Fig.S4 CUB induced cell death through autophagy-dependent pathways**

**(a)** Western blotting analysis of U87, U251 cells at 12h treatment of CUB (0μM, 20μM) or 3-MA(1mM) and CQ(30μM) pretreatment followed treatment of CUB(20μM). **(b)** Autophagy inhibitors suppressed U87 cell proliferation detected by the Cell-title Blue assay. Cells were incubated with 3-MA(1mM) or CQ (30μM) 12 h and then cultured in CUB(20μM) medium for the indicated times. Means ± SEM. *P < 0.05, **P < 0.01, ***P < 0.001

**
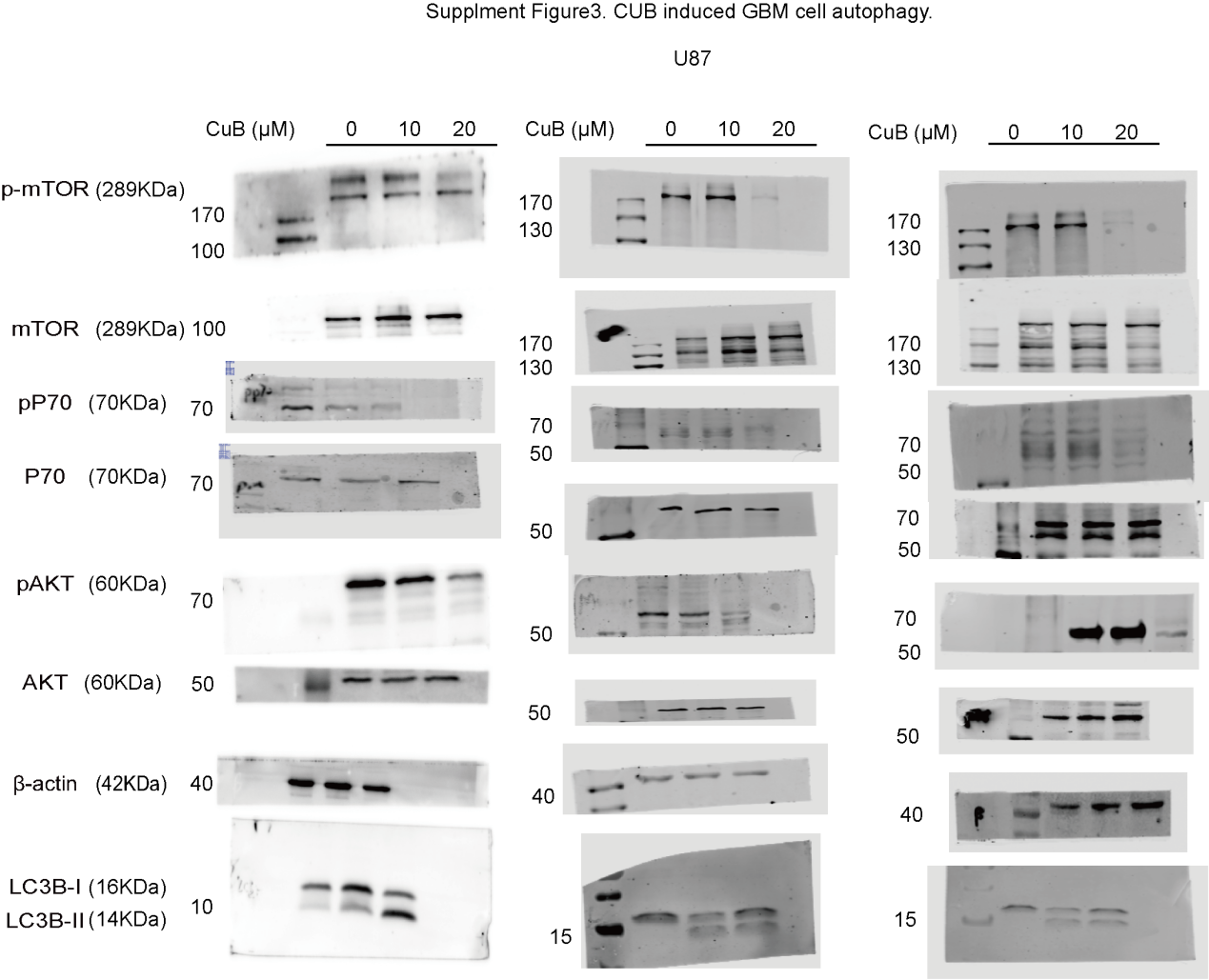
**

**Fig.S5 CUB induces GBM cell autophagy, blots origin images and replicates of Fig.3b(U87)**

**
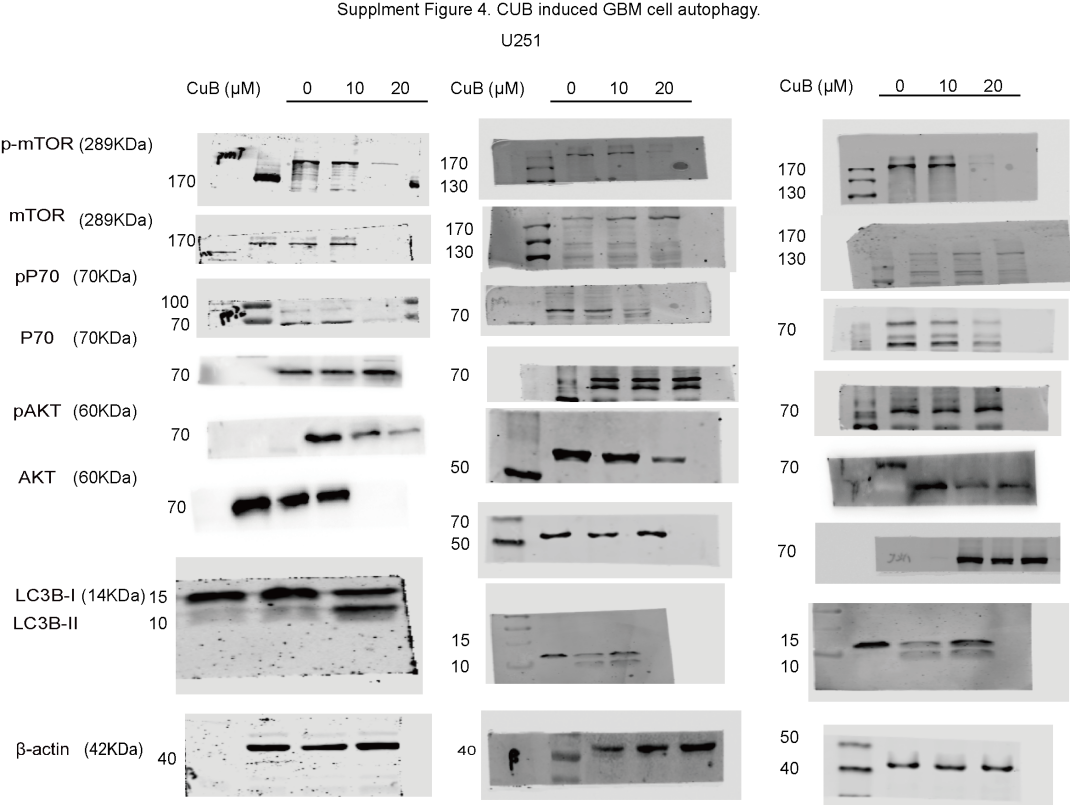
**

**Fig.S6 CUB induces GBM cell autophagy, blots origin images and replicates of Fig.3b(U251)**


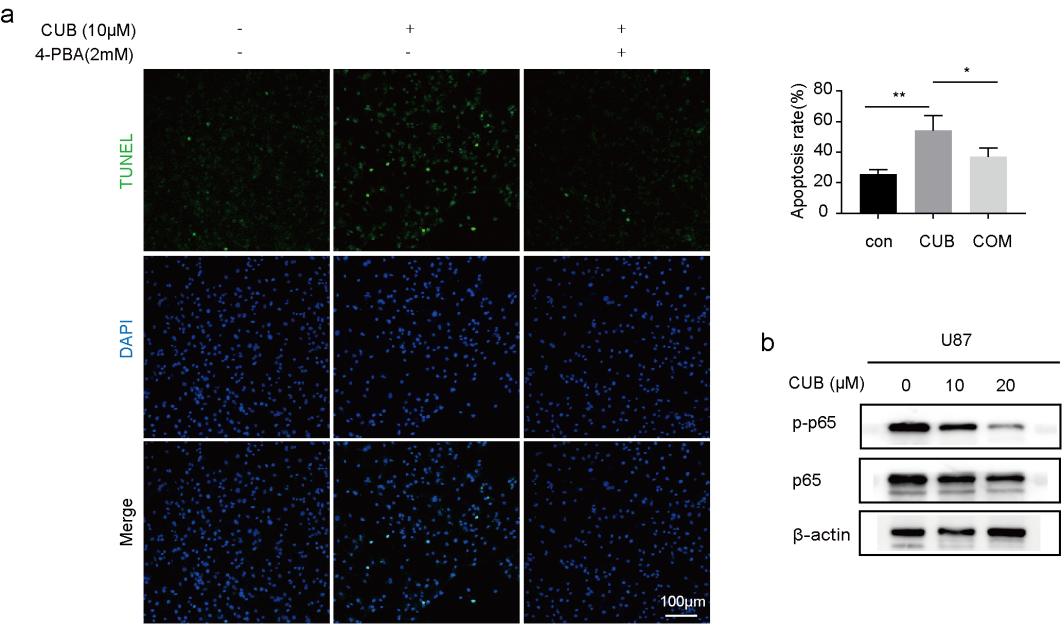


**Fig.S7 CUB promoted apoptosis and NF-κB activation in U87 cells**

1. Images of TUNEL staining Green: TUNEL-positive cells, Blue: DAPI. **(b)** Western Blot analysis of NF-κB pathway. All data are expressed as the mean ± SEM of values from experiments performed in triplicate. * P < 0.05, ** P < 0.01 and *** P < 0.001 compared to control (Cropped blots).

**
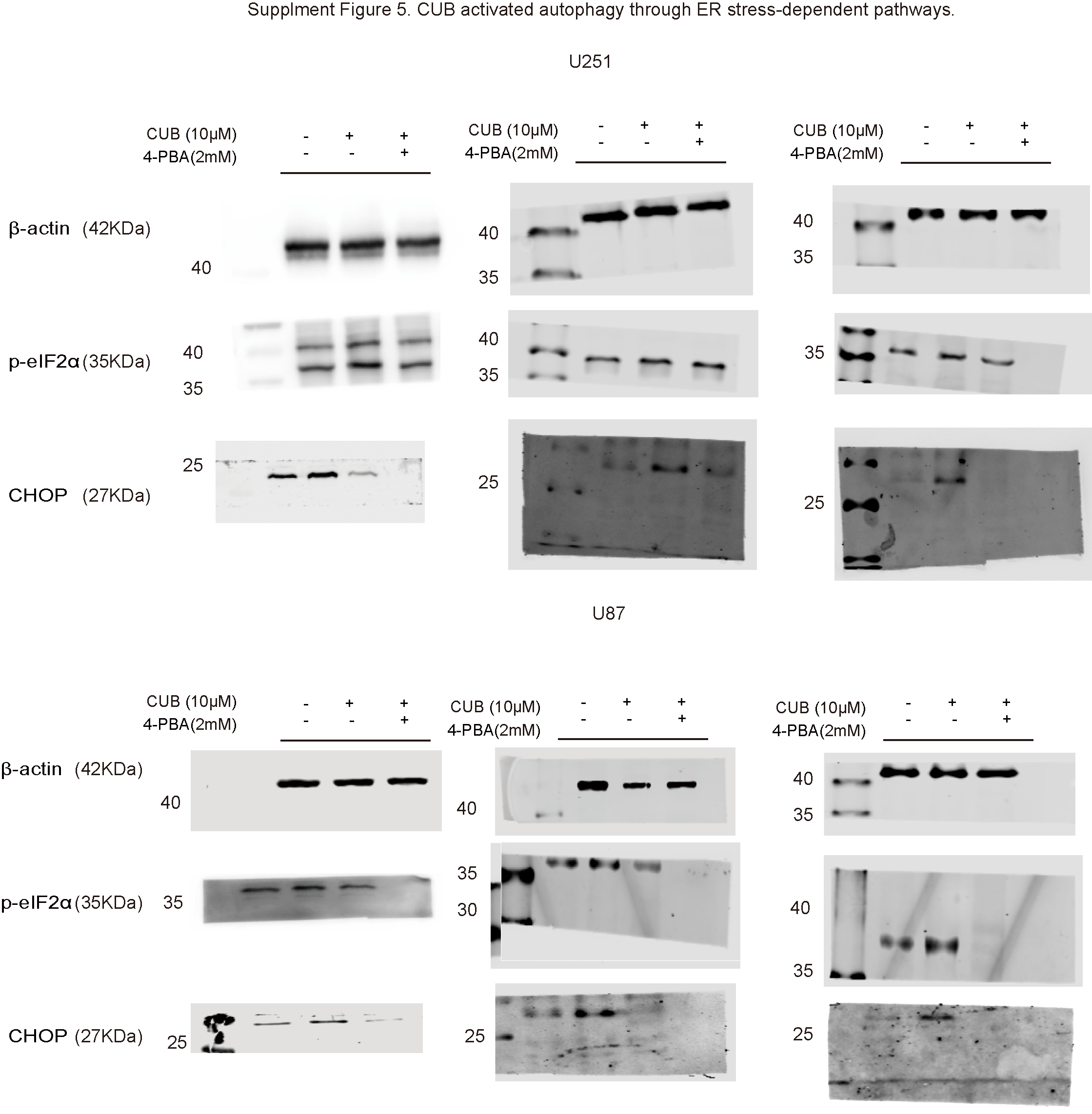
**

**Fig.S8 The ER stress inhibitor 4-PBA counteracts CUB, blots origin images and replicates of Fig.4a**

**
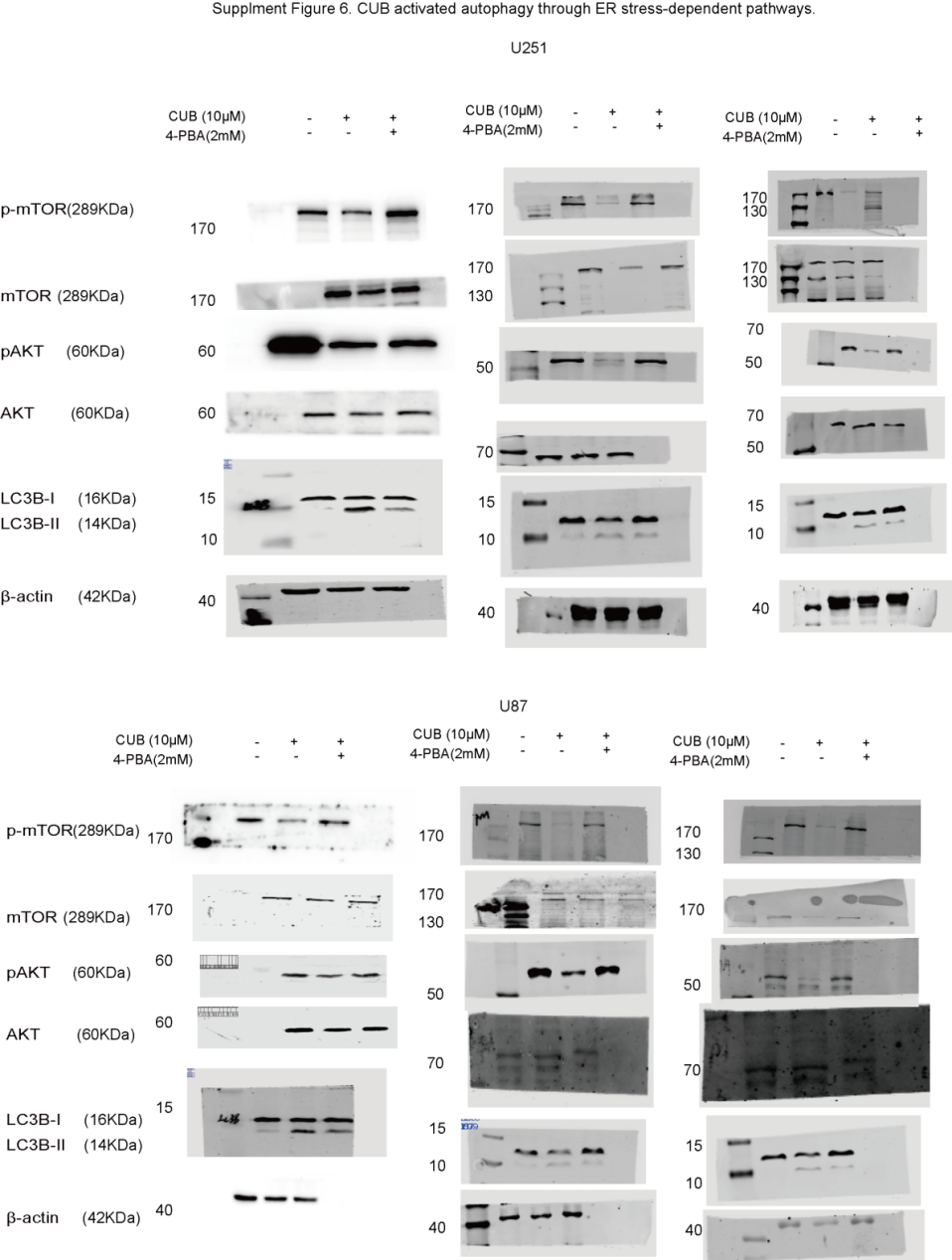
**

**Fig.S9 The ER stress inhibitor 4-PBA counteracts CUB, blots origin images and replicates of**

**Fig.4a**


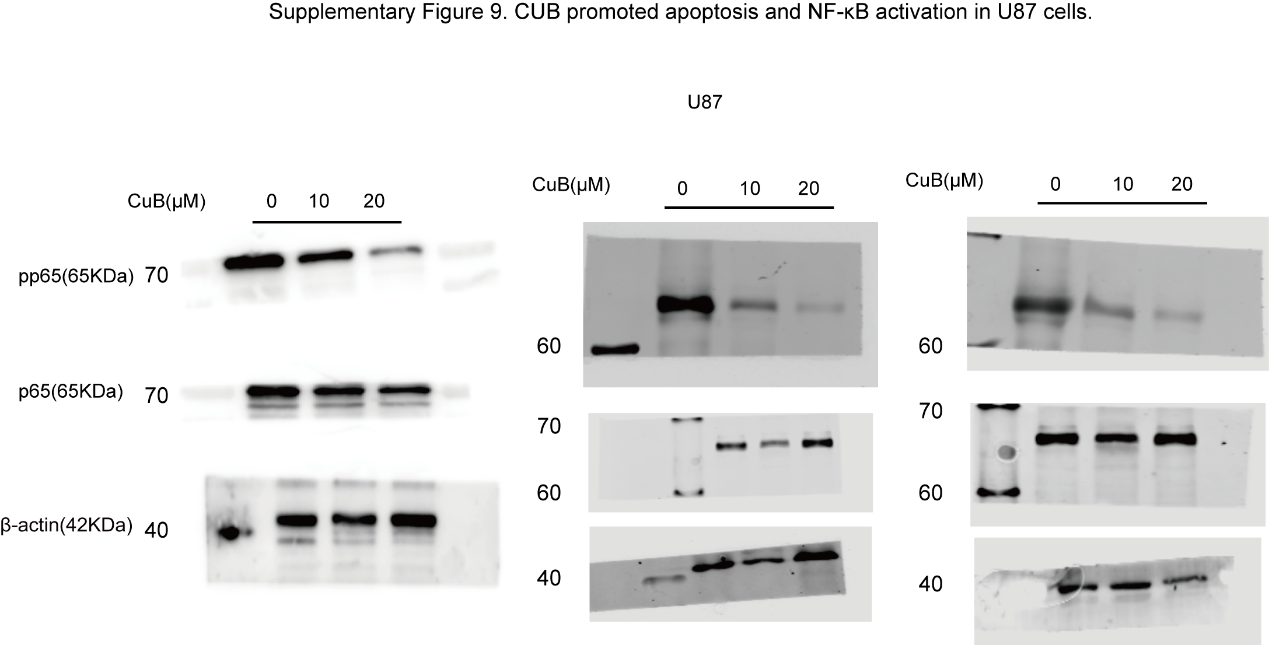


**Fig.S10 CUB promoted apoptosis and NF-κB activation in U87 cells. blots origin images and replicates of Fig.S1b**


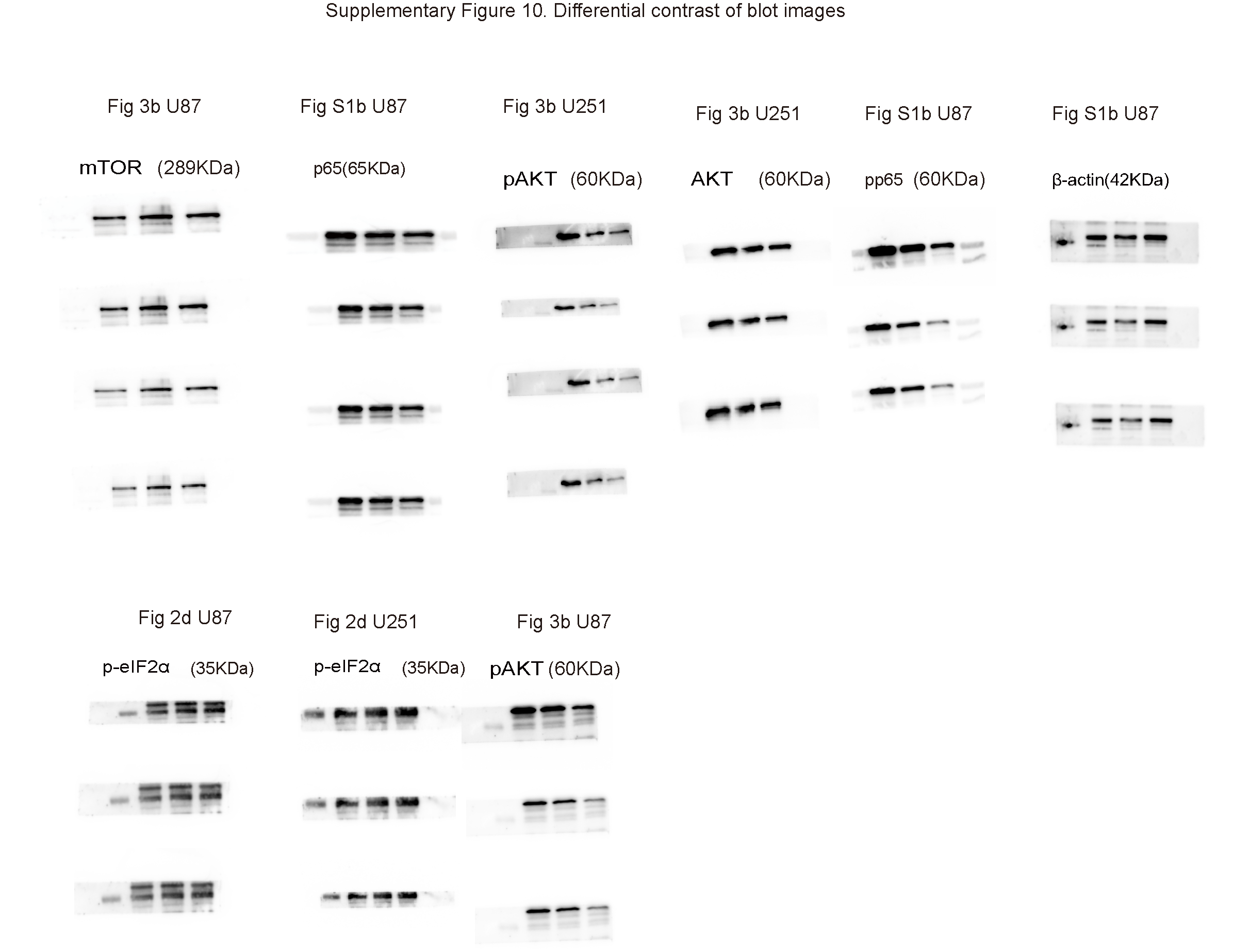


**Fig.S11 Part images of differential contrast blot**
